# Inoperable Endometrial Carcinoma Treated With External Beam Radiation and a Stereotactic Body Radiation Therapy Boost

**DOI:** 10.7759/cureus.81363

**Published:** 2025-03-28

**Authors:** Rohail Syed, Ramesh Boggula, Harriett S Jaenisch, Michael C Joiner, Steven R Miller

**Affiliations:** 1 Department of Oncology, Wayne State University School of Medicine, Detroit, USA

**Keywords:** conformal radiation therapy, early-stage endometrial cancer, gynec oncology, inoperable uterine cancer, morbidly obese

## Abstract

A significant comorbidity of endometrial cancer is severe obesity with a Body Mass Index (BMI) of greater than 40, which can dramatically limit treatment options such as surgery or brachytherapy. Alternative treatment options need to be investigated, and in this case report, we examine a female with severe obesity and a significantly enlarged uterus who was ineligible for surgery or intracavitary brachytherapy (ICBT). She subsequently underwent treatment with external beam radiation therapy (EBRT) alone to the pelvis, followed by a stereotactic radiation therapy boost (SBRT) to the uterus. Following treatment, her uterine bleeding subsided, and she is currently without evidence of disease.

## Introduction

Approximately 10% of early-stage endometrial carcinomas are inoperable due to a patient's elevated body mass index (BMI), anesthetic contradictions, and other comorbidities [1]. Also, approximately 57% of endometrial cancers can be linked to obesity [2], which can make patients ineligible for surgery and or intracavitary brachytherapy (ICBT). In cases of inoperable endometrial cancer, particularly for stage 1 and 2 diseases, with MRI evidence of deep myometrial invasion, the American Brachytherapy Society recommends that external beam irradiation (EBRT) be used in conjunction with ICBT [3]. However, due to our patient’s severe obesity (weight exceeding 200 kg), BMI greater than 60, as well as her greatly enlarged uterus of over 20 cm in size, ICBT was not a viable option. In this case report, we discuss a patient with inoperable endometrial cancer that was treated with EBRT to the pelvis and a stereotactic body radiation therapy (SBRT) boost to the uterus with curative intent.

## Case presentation

**Figure 1:**
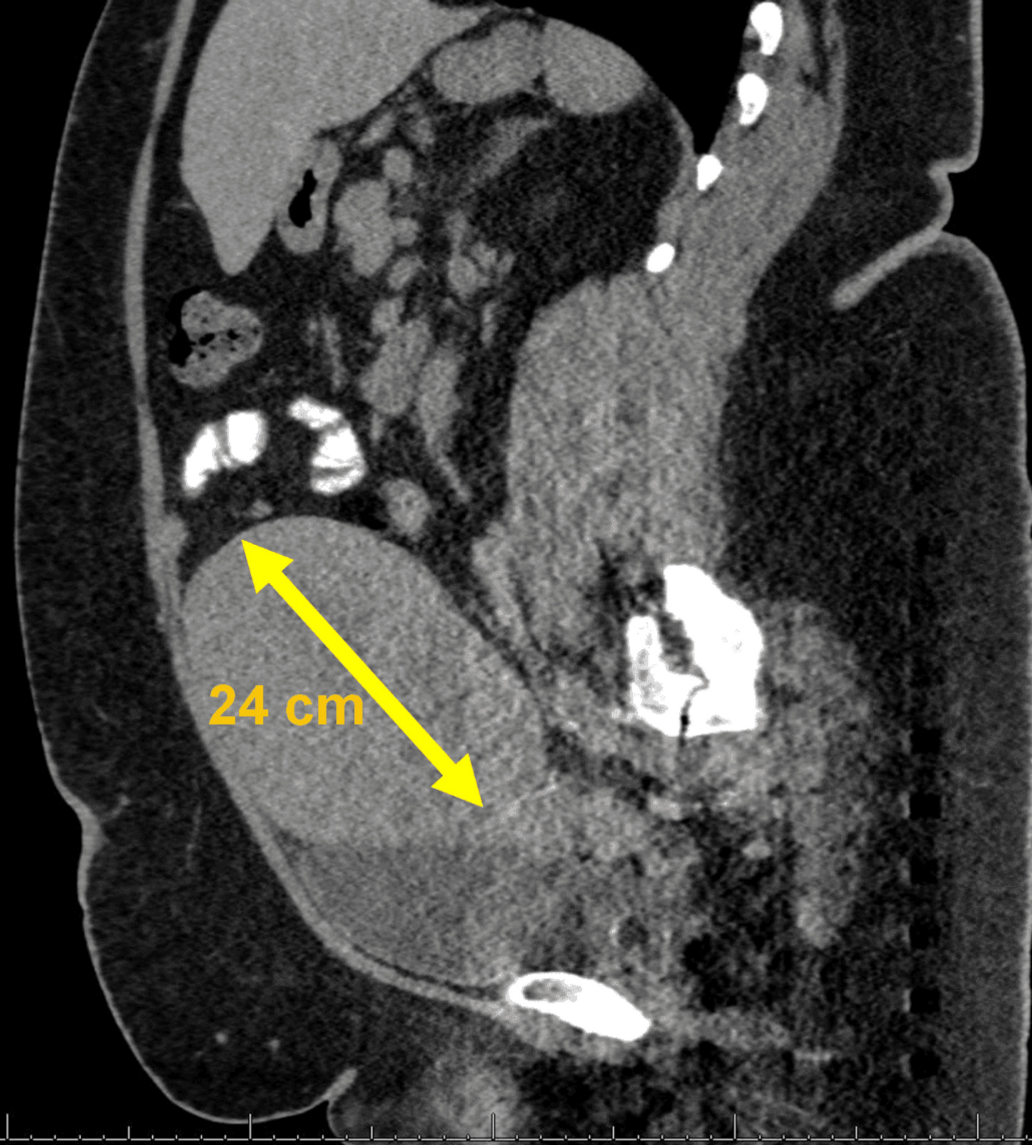
Sagittal view of a CT of the abdomen and pelvis The CT scan image reveals a uterus measuring approximately 24 cm in length.

A female in her mid-40s presented to her gynecologist with abnormal uterine bleeding for several months. She was subsequently admitted with a hemoglobin of 6 g/dL and underwent a transfusion of seven units of packed red blood cells. She was initiated on norethindrone acetate (Aygestin®), a progestin used to treat abnormal vaginal bleeding in adults, with a significant decrease in her vaginal bleeding. She also experienced intermittent abdominal cramping as well as nausea with the passing of blood clots vaginally. A pap smear revealed atypical glandular cells, and staging studies, including a CT scan of the abdomen and pelvis with contrast, showed an enlarged uterus measuring up to 24 cm in fundal length and 16.8 cm in the transverse dimension (Figure 1).

An endometrial biopsy was performed, which revealed an endometrioid endometrial carcinoma, International Federation of Gynecology and Obstetrics (FIGO) grade 1. Immunohistochemical stains displayed estrogen receptor-positive (estrogen-dependent endometrial tumors typically include grades 1 and 2 endometrioid adenocarcinomas) [4], p-53 (also known as Tumor protein P53) was wild-type. (TP53 mutation status, i.e., wild type, is associated with an unfavorable outcome) [5], and the mismatch repair gene was normal in this patient. Mismatch repair deficiency has also been associated with poor survival [6,7].

Secondary to the patient's body habitus (excessive weight and large size), she was unable to undergo a magnetic resonance imaging (MRI) scan of the pelvis, which is the preferred imaging modality for endometrial cancer since it can be used to evaluate the depth of myometrial invasion, cervical stromal involvement, extrauterine extent, and lymph node involvement [8]. A CT scan of the pelvis did not demonstrate any pelvic adenopathy, and the depth of myometrial invasion could not be evaluated using CT imaging. Her final stage was a clinical T1N0M0 endometroid endometrial carcinoma.

She was previously treated with Megace® (megestrol acetate), a progestin medication used to treat endometrial cancer, at a dose of 80 mg twice per day since she was not a surgical candidate. She has a history of severe obesity, weighing over 200 kg with  a BMI of greater than 60. She also has a history of congestive heart failure, insulin-dependent diabetes mellitus, hypertension, and HIV, managed with antiretrovirals with a recent cluster of differentiation 4 (CD4) count of 1424 and an undetectable viral load. 

Her case was discussed in a multidisciplinary tumor board, and the decision was made to treat her with primary radiation therapy since she was not a surgical candidate. She subsequently underwent a course of external beam radiation therapy (EBRT) to the pelvis with a stereotactic radiation therapy (SBRT) boost to the uterus. Prior to the initiation of treatment, she underwent a planning CT scan for radiation therapy planning. The clinical target volume for the pelvis (CTV pelvis) included the uterus, cervix, and proximal vagina with a 1 cm margin added, as well as the obturator, internal, external iliac, and common iliac lymph nodes. The planning target volume to treat the pelvis (PTV pelvis) included the CTV pelvis with a 1 cm margin in all directions. She underwent a course of EBRT and was treated to a dose of 4500 centigray (cGy) in 25 fractions to the PTV pelvis.

Due to the patient’s weight exceeding the weight limit of our brachytherapy treatment table, her overall size, and the size of her uterus (greater than 20 cm), she was not deemed a candidate for a high dose rate (HDR) brachytherapy boost. The size of our most extended HDR applicator was only 8 cm, which would not have been sufficient for treatment [3].

**Figure 2:**
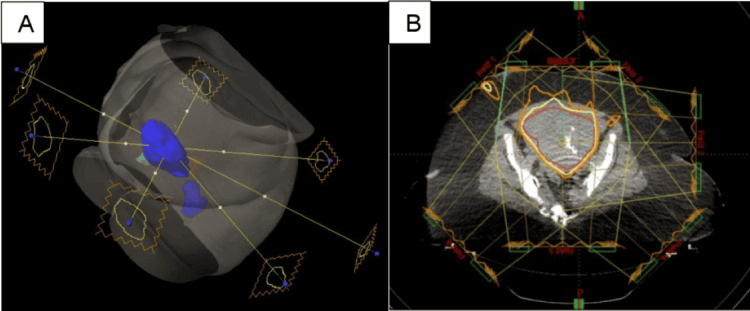
Radiation therapy plans applied to the patient (A) IMRT boost plan using a 7 field technique to treat the PTV boost volume. (B) Isodose distribution lines for 7 field SBRT plan. The PTV boost is displayed in red, the 95% isodose line is yellow, and the 80% isodose line is orange. SBRT: Stereotactic body radiation therapy; IMRT: Intensity-modulated radiation therapy; PTV: Planning target volume.

**Figure 3:**
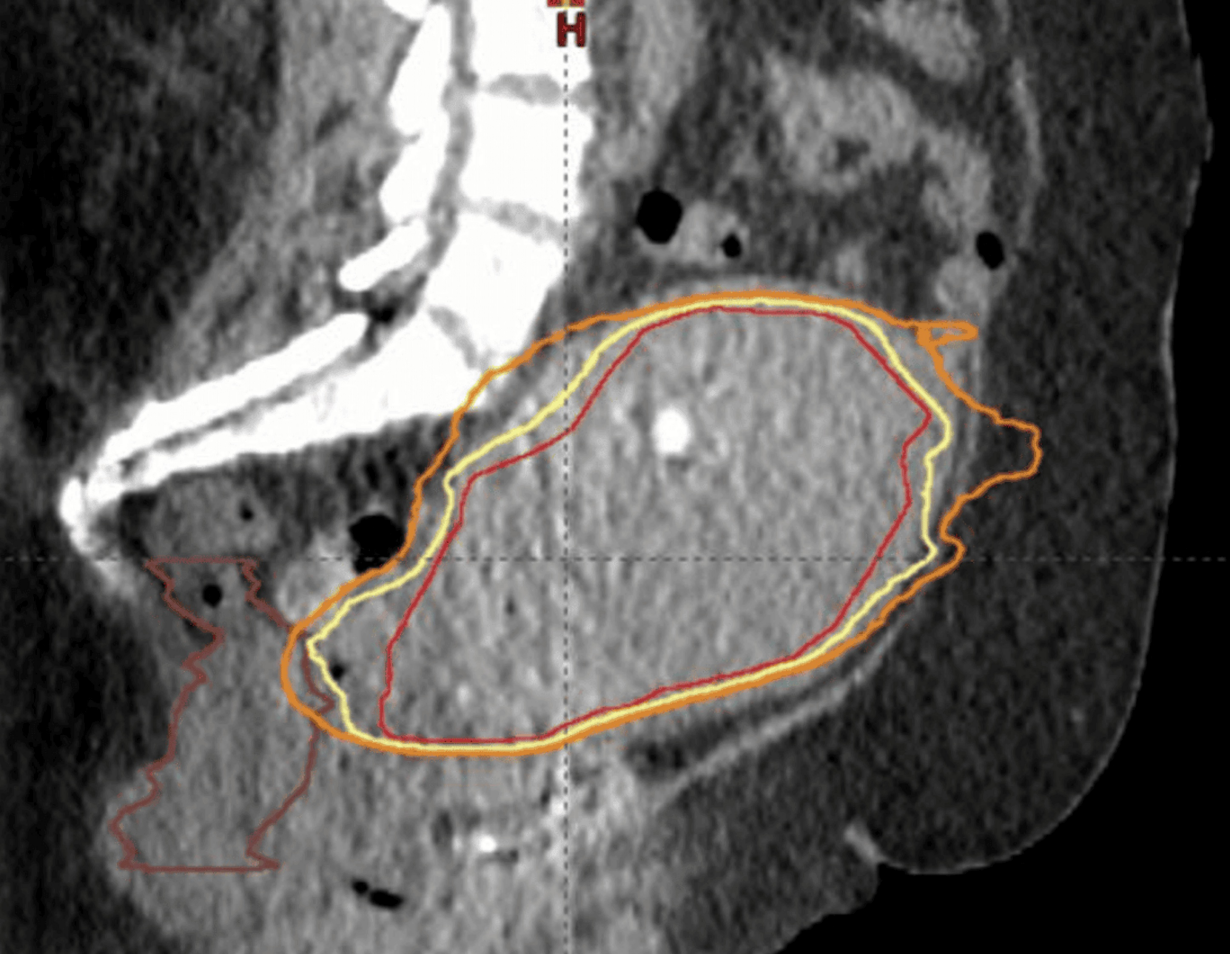
Sagittal view of stereotactic radiation therapy boost The Boost planning target volume (PTV) contour is displayed in red, the 95% isodose line is yellow, the 80% isodose line is orange, and the rectum is brown.

After the completion of 4500 cGy to the PTV pelvis, she underwent an SBRT to the uterus. The boost volume (PTV boost) consisted of the uterus contour with a 5 mm reduction in all dimensions to limit the dose to the small bowel, bladder, and rectum. Since an MRI could not be obtained and the disease could be visualized in the uterus, the goal was to treat as much of the uterus volume as possible without exceeding the dose limitations to the organs at risk (OARs). The PTV boost volume was treated to a total dose of 2000 cGy, in four fractions of 500 cGy per fraction. At least 95% of the PTV boost volume was covered by a 100% isodose line. (Figures 2, 3).

A cone beam CT scan was obtained before each treatment, and the positioning of the uterus was verified before each treatment. The patient was also treated with a full bladder to help with the reproducibility of the uterus position.

**Figure 4:**
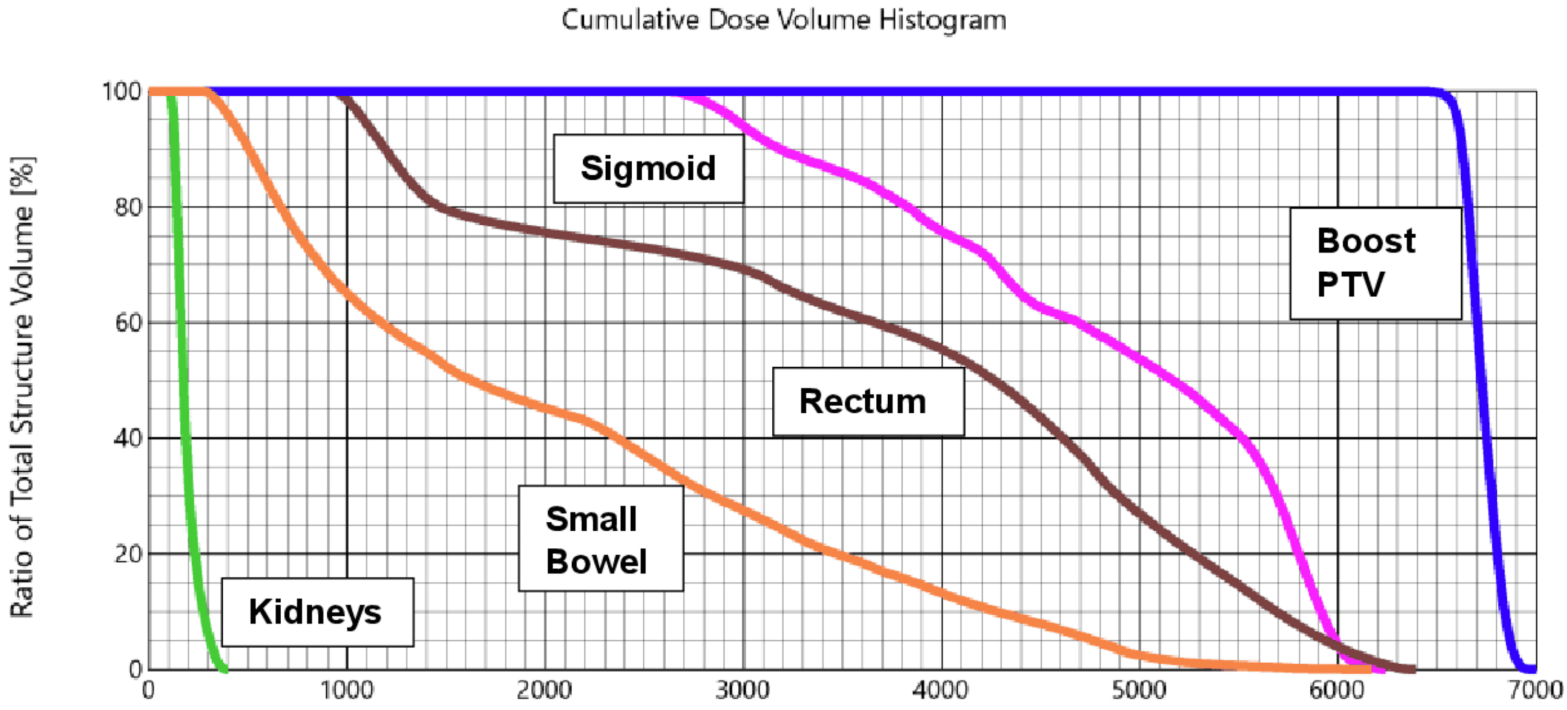
Dose volume histogram displaying the doses to the organs at risk Dark blue: Uterus; Magenta: Sigmoid colon; Green: Kidneys; Brown: Rectum; Orange: Small bowel.

She received a total of 4500 cGy to the PTV pelvis, followed by an SBRT boost to the PTV boost (uterus with a 5mm contour reduction) to an additional 2000 cGy in four fractions to a total dose of 6500 cGy to the PTV boost volume. The radiation doses to the at-risk organs are displayed in the Dose Volume Histogram (DVH) below (Figure 4).

The maximum dose to the uterus (dark blue) was 7000 cGy with a mean dose of 6730 cGy. The mean dose to the sigmoid colon (magenta) was 4846 cGy with a maximum of 6241 cGy; the right and left kidneys (green) were below 1800 cGy for a mean dose. The mean dose to the rectum (brown) was 3753 cGy with a maximum dose of 6393 cGy (goal of less than 7000 cGy), the maximum dose to the small bowel (orange) was 6169 cGy (goal of less than 6500 cGy) with a mean dose of 2059 cGy.

The patient tolerated the treatment well, with the resolution of her vaginal bleeding. She did experience some loose stools during treatment, but had no dysuria. At three months status post-treatment, the patient was seen by her primary care physician, and at that time, she had no complaints of vaginal bleeding. The patient underwent a follow up at three and six months status post treatment with no reported vaginal bleeding or discharge. Unfortunately, she was lost to further follow-up.

## Discussion

At this time, there are limited studies available examining the treatment of inoperable uterine cancer with external beam radiation (EBRT) alone. However, the available data may suggest that this may be a viable option. A study by Podzielinski et al. retrospectively reviewed 101 endometrial cancer patients aged 39-94 years who could not undergo surgery due to medical issues. All patients had clinical stage I disease, with the majority (82%) showing cancer invading less than half of the myometrium, while the remaining patients showed no myometrial invasion. Radiation therapy consisted of EBRT and intracavitary radiation therapy (ICRT (61%), ICRT alone (26%), EBRT plus ICRT (10%), and EBRT alone (4%). The five-year disease-free survival (DFS) and overall survival (OS) rates were >80%. Of the three patients treated with pelvic radiation alone, none experienced a disease recurrence [9].

A retrospective review by Olson et al. examined 32 women with inoperable biopsy-proven endometrial cancer treated with definitive radiation. All patients received EBRT to the pelvis, followed by a high dose rate (HDR) brachytherapy, or an intensity-modulated radiation therapy (IMRT)/conformal boost. The initial pelvic field was treated with a four-field technique to a median dose of 4500 cGy. Twenty-four patients were treated with an HDR brachytherapy boost consisting of 1700 cGy in two fractions, and eight patients received an external beam radiation boost with a median boost dose of 2300 cGy. The external beam radiation boost volume comprised the uterus with a 1-2 cm expansion. The most common comorbidity was obesity, with a median BMI of 39.9. A significant difference in the overall survival rate, disease-specific survival, or side effects from treatment among the different treatment modalities was not identified [10]. The most common acute side effects included diarrhea (16%) and rectal bleeding (3%). These side effects were not different between the IMRT/conformal and HDR brachytherapy groups [10]. The authors of this study concluded that EBRT treatment could match the efficacy of treatment with HDR brachytherapy without an increase in the severity or frequency of side effects.

An additional study conducted by Tsujii et al. examined 19 patients diagnosed with uterine carcinoma who were treated with EBRT to the pelvic lymph nodes to a dose of 5040 cGy in 28 fractions [11]. After 3000-4000 cGy was delivered to the pelvic lymph nodes, the central target volume (uterus), as determined by CT scans, was shielded. The central target volume was then boosted with proton therapy with doses ranging between 4600 and 6300 cGy (mean 5800 cGy) with a daily fraction size of 260-380 cGy (mean 330 cGy). A total combined dose consisting of the EBRT plus the proton therapy was 7900-9200 cGy (mean 8500 cGy) to the uterus. Two patients with stage IIIb disease experienced local failures, and none of the stage IIb patients experienced a failure. Most patients tolerated the treatment well, although two patients developed grade 3 radiation-related proctitis. The actuarial survival rate at 3 years was 87.5% for Stage IIb patients and 75.8% for Stage IIIb patients. Median survival was 38 months [11]. 

Finally, a study by Kemmerer et al. evaluated eleven patients with stage I-III endometrial cancer who were not considered candidates for hysterectomy or intracavitary brachytherapy secondary to comorbidities (91%) or refusal to undergo either procedure (9%) [12]. The patients were initially treated with EBRT with a median dose of 4500 cGy in 25 fractions, followed by an SBRT boost with a median dose of 3000 cGy in five treatments delivered twice weekly. The SBRT boost volume was defined as a 1-cm expansion around the endometrial cavity and any radiographically identified tumor mass. At 12 and 18 months, the overall freedom from disease progression for the patients treated with EBRT and an SBRT boost was 68% and 41%. Stage IA endometrial carcinoma patients had an overall freedom from progression of 100%, and stage IB patients had an overall freedom from progression of 33% at 18 months. All patients achieved cessation of vaginal bleeding after the treatment, and the overall survival for the whole group was 57% at 18 months [12]. 

When reviewing the results of radiation and brachytherapy for early-stage endometrial cancer, a single-institution retrospective analysis by Shen et al. examined 55 medically inoperable patients with biopsy-proven endometrial carcinoma who were treated with definitive radiation therapy [13]. Forty-nine patients (89%) were treated with HDR brachytherapy and EBRT, and six patients (11%) were treated with HDR brachytherapy alone. The two-year overall survival in patients with low-risk endometrial carcinoma (uterine-confined grade 1-2 endometrioid adenocarcinoma) was 92%, and the two-year cancer-free survival was 82% [13].

When comparing outcomes of brachytherapy alone versus external beam irradiation and brachytherapy, a study by the American Brachytherapy Society evaluated survival outcomes according to the type of radiation therapy for stage 1 endometrial cancer from studies over different periods of time. In summary, the five-year cancer-specific survival of patients with stage I endometrial cancer treated with only ICRT (either low or high-dose) or with additional EBRT ranged from 65-85% and up to 95%, respectively. The survival rates with only ICRT or additional EBRT were 40-69% and 88% for stage II disease and 14% and 57% for stage III disease [3,14].

When considering radiation and/or brachytherapy, a study by Gill et al. reviewed 853 stage 1 endometrial cancer patients treated with EBRT, brachytherapy, and a combination of the two [15]. Three hundred fifty-two patients received EBRT with curative intent, with a mean dose of 4920 cGy. Thirty-three patients received palliative EBRT with a mean dose of 1910 cGy. The median survival rate for EBRT with curative intent alone was 31.5 months. The median survival rate for brachytherapy alone and EBRT plus brachytherapy was higher at 44.6 months and 57.1 months. The median survival rate for palliative EBRT was much lower at 12.1 months [15]. The authors concluded that this data may suggest that brachytherapy in tandem with EBRT is generally the most effective treatment option; EBRT alone is an effective treatment that can significantly increase the overall survival rate.

Examining surgery alone for early-stage endometrial cancer, a study by Rahatli et al. retrospectively reviewed 62 patients with stage IB endometrial cancer [16]. These patients were initially treated surgically by the same surgeon with comprehensive staging, including a total abdominal hysterectomy, bilateral salphingooopherectomy, bilateral pelvic and paraaortic lymph node dissection, and omentectomy. The majority of the patients had grade 1-2 disease. Thirteen patients (21%) received intravaginal brachytherapy (IVBT), and one received EBRT. Three patients experienced disease recurrence (4.8%). The relapse-free survival at five years was 94.4%, and overall survival was 93.1%. Patients with stage IB disease had a relatively low recurrence rate, and the study suggested that surgery alone without adjuvant radiation may be sufficient for many patients with this stage of endometrial cancer [16].

In addition, a study by Eltabbakh and Moore examined 48 women with stage II endometrial cancer treated with surgical intervention [17]. The estimated five-year overall survival rate was 92.1%, with a disease-free survival rate of 89.9%. None of the 20 patients treated with a total abdominal hysterectomy followed by whole pelvic radiation and vaginal cuff radiation therapy experienced recurrent disease. In contrast, three out of 17 patients who were treated by total abdominal hysterectomy followed by either whole pelvic radiation or vaginal cuff therapy alone experienced recurrence. The authors stated that this data suggests that external beam radiation therapy and vaginal cuff brachytherapy used as adjuvant treatment are very effective in treating endometrial cancer [17].

## Conclusions

External beam radiation and intracavitary brachytherapy or intracavity brachytherapy alone are the standard of care when treating inoperable endometrial cancer. Intracavitary brachytherapy, though, is not always an option in some obese and medically unfit patients. The treatment of these patients with external beam radiation and a stereotactic brachytherapy boost to the uterus is a possible alternative treatment option with promising data. It appears, from reviewing the literature, that external beam radiation and a stereotactic radiation boost to the uterus can be a treatment option for inoperable early-stage endometrial carcinoma. Additional research will need to be performed to examine this subject.
